# Male-female differences in thoracic aortic diameters at presentation of acute type A aortic dissection

**DOI:** 10.1016/j.ijcha.2023.101290

**Published:** 2023-10-29

**Authors:** F. Meccanici, A.W. Bom, W.G. Knol, A.L. Gökalp, C.G.E. Thijssen, J.A. Bekkers, G.S.C. Geuzebroek, M.M. Mokhles, R.R.J. van Kimmenade, R.P.J. Budde, J.J.M. Takkenberg, J.W. Roos-Hesselink

**Affiliations:** a Department of Cardiology, Erasmus University Medical Centre, Rotterdam, Netherlands; b Department of Radiology and Nuclear Medicine, Erasmus University Medical Centre, Rotterdam, Netherlands; c Department of Cardiothoracic Surgery, Erasmus University Medical Centre, Rotterdam, Netherlands; d Department of Cardiology, Radboud University Medical Centre, Nijmegen, Netherlands; e Department of Cardiothoracic Surgery, Radboud University Medical Centre, Nijmegen, Netherlands; f Department of Cardiothoracic Surgery, Utrecht University Medical Centre, Utrecht, Netherlands

**Keywords:** Thoracic aortic dissection, Stanford type A dissection, Aortic dimensions, Sex, Gender

## Abstract

**Background:**

Acute type A aortic dissection (ATAAD) is a highly lethal event, associated with aortic dilatation. It is not well known if patient height, weight or sex impact the thoracic aortic diameter (TAA) at ATAAD. The study aim was to identify male–female differences in TAA at ATAAD presentation.

**Methods:**

This retrospective cross-sectional study analysed all adult patients who presented with ATAAD between 2007 and 2017 in two tertiary care centres and underwent contrast enhanced computed tomography (CTA) before surgery. Absolute aortic diameters were measured at the sinus of Valsalva (SoV), ascending (AA) and descending thoracic aorta (DA) using double oblique reconstruction, and indexed for body surface area (ASI) and height (AHI). Z-scores were calculated using the Campens formula.

**Results:**

In total, 59 % (181/308) of ATAAD patients had CT-scans eligible for measurements, with 82 female and 99 male patients. Females were significantly older than males (65.5 ± 12.4 years versus 60.3 ± 2.3, p = 0.024). Female patients had larger absolute AA diameters than male patients (51.0 mm [47.0–57.0] versus 49.0 mm [45.0–53.0], p = 0.023), and larger ASI and AHI at all three levels. Z-scores for the SoV and AA were significantly higher for female patients (2.99 ± 1.66 versus 1.34 ± 1.77, p < 0.001 and 5.27 [4.38–6.26] versus 4.06 [3.14–5.02], p < 0.001). After adjustment for important clinical factors, female sex remained associated with greater maximal TAA (p = 0.019).

**Conclusion:**

Female ATAAD patients had larger absolute ascending aortic diameters than males, implying a distinct timing in disease presentation or selection bias. Translational studies on the aortic wall and studies on growth patterns should further elucidate these sex differences.

## 1 Introduction

Acute type A aortic dissection (ATAAD) is an alarming cardiovascular event carrying a high mortality risk [1], [2]. The incidence of ATAAD is estimated at 2–4 per 100.000 annually [3], [4] and common risk factors include hypertension, smoking and hyperlipidaemia [4], [5]. The diameter of the thoracic aorta is strongly associated with the risk of ATAAD and rupture [6]. Therefore, guidelines recommend preventive surgery for patients with an absolute thoracic aortic diameter of ≥55 mm, while lower thresholds are used for specific diagnoses such as Marfan syndrome or other heritable thoracic aortic diseases (HTAD). Rapid aortic growth and additional risk factors are also important to take into account [7], [8].

In light of patient-specific care, it can be questioned whether the absolute diameter is the most adequate parameter to use for surgical indication, since previous studies have consistently found that the thoracic aortic diameter is positively associated with age and body size [9], [10], [11]. Most guidelines use the absolute diameter, although for instance in women with Turner syndrome, correction for body surface area (BSA) is advised [12]. Furthermore, male–female differences have been observed in both absolute aortic diameters and aortic growth [13], [14], [15]. For abdominal aortic aneurysms, a lower threshold for diagnosis or treatment in females has been suggested [16]. However, in current guidelines on thoracic aortic disease little attention is paid to male–female differences and discussions on indexing the aorta diameter are ongoing [7], [8]. Although the diameter at ATAAD might be larger in the acute phase than prior to dissection [17], [18], comparing males and females and comparing absolute versus indexed-diameters will shed light on this discussion.

The aim of this study was to determine male–female differences in absolute and indexed aortic diameters at presentation of ATAAD.

## 2 Methods

### 2.1 Study design and study population

A retrospective cross-sectional study was performed including all adult patients (≥18 years old) from two tertiary care centres, who presented with an acute type A aortic dissection (ATAAD) in the period between 2007 and 2017 and underwent contrast enhanced computed tomography at the time of presentation. Participating study centres were the Erasmus Medical Centre Rotterdam and Radboud University Medical Centre Nijmegen. The study was approved by the local Medical Ethical Committee (MEC-2018-1535) and was performed according to local and international practice guidelines.

### 2.2 Endpoints

The primary objective was to examine male–female differences in absolute, BSA- and height-indexed aorta diameters at three pre-defined levels of the thoracic aorta: (1) the Sinus of Valsalva (SoV); (2) ascending aorta (AA); and (3) descending aorta (DA). In addition, the maximal thoracic aortic diameter (ADmax) was assessed. Secondary objectives were to calculate Z-scores for the SoV and AA diameters, to study sex-specific distributions of the absolute, BSA- and height-indexed thoracic aortic diameters, associations between patient characteristics and the maximal thoracic aortic diameter and the association between sex and the thoracic aortic diameter after adjustment for important clinical characteristics.

A sample size calculation was performed based on an estimated difference in ascending aortic diameters between males and females in the general Dutch population [19] using a web-based calculator [20], resulting in a required total sample size of 60 patients (Appendix I).

### 2.3 Data collection and definitions

Data were collected in an anonymized standardized case report form using OpenClinica (OpenClinica, LLC, version 3.6). Patient characteristics were collected retrospectively using patient files. All included variables with their definitions are depicted in Appendix II. Acute was defined as presentation within 14 days after symptom onset [7], [21]. The body surface area (BSA) was calculated using the Du Bois method [22]. BSA- and height-indexed aortic diameters were calculated using the following formulas: aortic size index (ASI) = aortic diameter (mm)/BSA (m^2^) and aortic height index (AHI) = aortic diameter (mm)/body height (m). Z-scores were calculated for the SoV and AA using the Campens formula, taking into account patients’ BSA, sex, and age [23].

### 2.4 Imaging

**Fig. 1:**
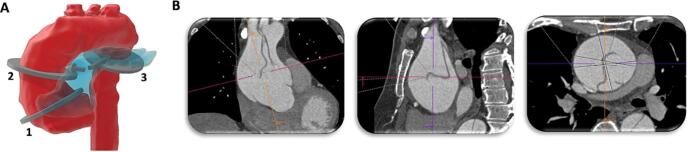
Double-oblique measurement method for thoracic aortic diameters. Panel A: The circular planes, (1) Sinus of Valsalva, the (2) ascending and (3) descending aorta, are schematically illustrated in the aorta (red). The arrows illustrated with the second and third plane demonstrate the cross-section of the midpoint of the pulmonary artery (blue) with the aorta in the frontal plane, which was used to determine respectively the ascending and descending aorta. Panel B: These images demonstrate the aortic diameters of the ascending aorta (plane 2), respectively representing the frontal plane, the sagittal and the axial plane until perpendicular to the axis of the aorta. The maximum diameter was obtained by means of multiplane reconstruction using the double oblique method. These three steps were performed for the measurement at the Sinus of Valsalva, the ascending and descending aorta separately. (For interpretation of the references to colour in this figure legend, the reader is referred to the web version of this article.)

Contrast enhanced cardiac tomography (CT) scans were retrospectively collected at ATAAD presentation before surgical treatment. The aortic diameters were measured at three locations: the sinus of Valsalva (SoV) (using the cusp to commissure method), the ascending thoracic aorta (AA) and the descending thoracic aorta (DA). The latter two locations were consistently measured at the axial level where the pulmonary artery crosses the aorta in the sagittal plane. The maximum diameter was obtained by means of multiplanar reconstruction using the double oblique method, as visualized in Fig. 1. The outer-edge to outer-edge method was used in order to include the aortic wall and hematoma [24]. The maximal thoracic aortic diameter (ADmax) indicated the largest diameter that was measured*.*

### 2.5 Statistical analysis

Data analysis was performed using statistical and computing programme R (version 3.4.3) and SPSS statistics (IBM SPSS Statistics version 28.0.). Normally distributed continuous data were presented as the mean and standard deviation and compared between males and females using a Students *t*-test. Skewed continuous data were presented as median and interquartile range and compared between males and females using the Mann-Whitney *U* test. Categorical data were presented as percentages or frequencies and compared between males and females using the chi-square test or the Fisher exact test as appropriate. Data distribution was checked using the Shapiro-Wilk test. To ensure transparent reporting of data, missing values in the patient characteristics were reported. Sex-specific distributions with median, 90th and 95th percentile of absolute, BSA- and height-adjusted aortic diameters were calculated.

Associations between patient characteristics and absolute maximal thoracic aortic diameters (ADmax) were quantified with univariable and multivariable linear regression analysis. A multivariable model was constructed to study the association between sex and the absolute maximal thoracic aortic diameter adjusting for pre-specified clinically relevant variables based on previous literature: age, history of hypertension and known thoracic aortic aneurysm [25]. As additional analysis influential observations were removed in studying the effect of sex on the ADmax. The maximal thoracic aortic diameter was log2-transformed in order to satisfy the linearity assumption. The missing data pattern was assumed to be missing at random and missing values were imputed with multiple imputation using the *mice* package with 5 imputed datasets and 10 iterations. Variables were imputed up to a 15 % rate of missing values.

Two sensitivity analyses were performed: (1) comparing the baseline characteristics of patients with and without an available contrast-enhanced CT-scan available (2) aortic diameter measurements excluding patients with known bicuspid aortic valves or heritable thoracic aortic disease (HTAD).

A two-sided P-value < 0.05 indicated statistical significance.

## 3 Results

### 3.1 Patient and imaging characteristics

**Fig. 2:**
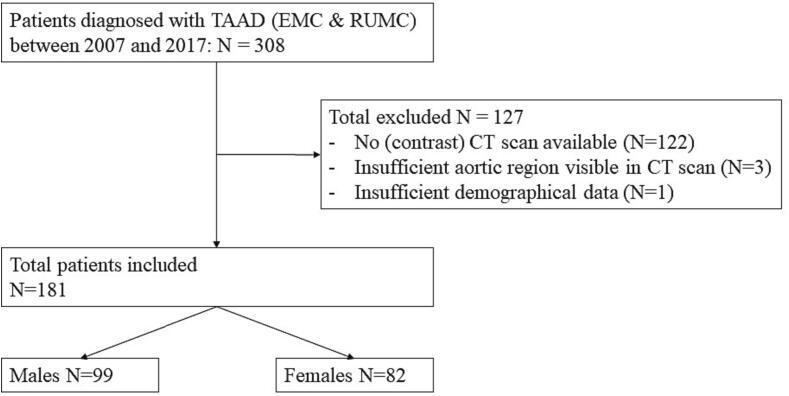
Flowchart of patient selection. TAAD: Acute type A aortic dissection; EMC: Erasmus MC; RUMC: Radboud University Medical centre; CT: computed tomography.

In Fig. 2 the flowchart of the patient selection is shown. In total 308 patients were diagnosed with ATAAD in the two participating centres, of whom 181 (59 %) had an eligible CT-scan. In Table 1 the patient and imaging characteristics are depicted for the total study population (n = 181) and males (n = 99) and females (n = 82) separately. Female patients were significantly older than male patients (66 ± 12 years versus 60 ± 12 years, p = 0.024). Furthermore, females had significantly lower height and body surface area (BSA) (1.7 ± 0.1 m versus 1.8 ± 0.1 m, p < 0.001 and 1.8 ± 0.2 mm/m^2^ versus 2.1 ± 0.2 mm/m^2^, p < 0.001).

**Table 1:** Patient and imaging characteristics.

	Total (n = 181)	Males (n = 99)	Females (n = 82)	P-value	Missing n (%)
*Patient demographics*					
**Age (years)**	62.2 ± 12.5	60.3 ± 12.3	65.5 ± 12.4	**0.024**	0 (0)
**Height (m)**	1.6 ± 0.1	1.8 ± 0.1	1.7 ± 0.1	**<0.001**	2 (1)
**Weight (kg)**	80.3 ± 15.2	88.4 ± 12.7	70.5 ± 11.9	**<0.001**	3 (2)
**BSA (m^2^)**	2.0 ± 0.2	2.1 ± 0.2	1.8 ± 0.2	**<0.001**	3 (2)
**BMI (kg/m^2^)**	25.6 [23.4–28.1]	26.3 [24.1–29.3]	24.4 [22.7–27.1]	**0.005**	3 (2)
**History of hypertension (%)**	99 (55)	48 (49)	51 (62)	0.061	2 (1)
**Hyperlipidaemia (%)**	25 (15)	11 (11)	14 (17)	0.211	8 (4)
**Diabetes mellitus (%)**	3 (2)	2 (2)	1 (1)	0.662	5 (3)
**COPD (%)**	14 (8)	5 (5)	9 (11)	0.136	2 (1)
**History of CVA (%)**	10 (6)	4 (4)	6 (7)	0.352	2 (1)
**History of MI (%)**	7 (4)	4 (4)	3 (4)	1.000	2 (1)
**Chronic kidney disease (%)**	3 (2)	0 (0)	3 (4)	0.089	3 (2)
HTAD (%)*	5 (3)	2 (2)	3 (4)	0.660	0 (0)
**Known TAA prior to presentation (%)**	19 (11)	7 (7)	12 (15)	0.091	3 (2)
**Smoking (%)**				0.335	73 (40)
**Never**	41 (38)	20 (20)	21 (26)		
**Currently**	45 (42)	29 (29)	16 (20)		
**Past**	22 (20)	13 (13)	9 (11)		
**Prior cardiac surgery (%)**				0.191	1 (1)
**None**	172 (95)	93 (93)	79 (96)		
**CABG**	4 (2)	3 (3)	1 (1)		
**PCI**	2 (1)	0 (0)	2 (2)		
**Other**	2 (1)	2 (2)	0 (0)		
**Prior aortic surgery (%)**	5 (3)	3 (3)	2 (2)	0.649	4 (2)
**Prior dissection or aneurysm in other major artery (%)**	5 (3)	2 (2)	3 (4)	0.661	1 (1)
**BAV (%)**	6 (4)	2 (2)	4 (5)	0.413	10 (6)
**History of AS (%)**	2 (3)	0 (0)	2 (2)	0.493	111 (61)
**History of AR (%)**	4 (6)	2 (2)	2 (2)	1.000	111 (61)
*CT-imaging characteristics*					
**Contrast-enhanced**	181 (1 0 0)	99 (1 0 0)	82 (1 0 0)	–	0 (0)
**ECG-triggered**	102 (56)	56 (56)	46 (56)	0.950	0 (0)
**Dissected SoV**	181 (54)	57 (58)	40 (49)	0.273	1 (1)
**Dissected AA**	181 (95)	93 (94)	79 (96)	0.515	0 (1)
**Dissected DA**	181 (71)	66 (67)	62 (76)	0.188	0 (1)

Normally distributed continuous variables are expressed as mean ± SD, skewed continuous variables are expressed as median and 25th-75th percentile, and categorical values are expressed as percentages. P-values < 0.05 are depicted in bold. *Heritable thoracic aortic disease as diagnosed before or after ATAAD presentation, including one female with Marfan syndrome, one female with Loeys-Dietz syndrome, one female with ACTA2 mutation, and two males with other genetic diseases.

Abbreviations: BMI: body mass index; BSA: body surface area; MI: myocardial infarct; HTAD: heritable thoracic aortic disease; TAA: thoracic aortic aneurysm; CABG: coronary artery bypass graft; PCI: percutaneous coronary intervention; BAV: bicuspid aortic valve; AS: aortic valve stenosis; AR: aortic regurgitation; CT: cardiac tomography; ECG: electrocardiography; SoV: Sinus of Valsalva, AA: ascending aorta, DA: descending aorta.

In Appendix III patients with CT-scan (n = 181) were compared with patients without available CT-scan (n = 127) with regard to patient characteristics, which showed no significant differences

### 3.2 Thoracic aortic diameters

Table 2 demonstrates the absolute, aortic size indexed (ASI) and aortic-height indexed (AHI) diameters of the sinus of Valsalva (SoV), ascending (AA), descending aorta (DA) and maximal thoracic aortic diameter (ADmax) for both males and females. For the total study population (n = 181), the median and mean ADmax were 50.0 [47.0; 56.0] mm and 52.0 ± 8.7 mm, respectively. The absolute aortic diameters were significantly larger in females when compared to males at the level of the AA and ADmax (51.0 [47.0; 57.0] mm versus 49.0 [45.0; 53.0] mm, p = 0.02 and 52.0 [47.0; 57.0 mm versus 49.0 [46.0; 54.0] mm, p = 0.027). In addition, females had greater BSA-indexed and AHI-indexed diameters in all three regions and ADmax when compared to males (Table 2). When excluding patients with bicuspid aortic valve or HTAD (n = 170), the results were comparable to the values in the total study population (Appendix IV).

**Table 2:** Absolute, BSA-indexed and height-indexed diameters of the Sinus of Valsalva, ascending aorta, descending aorta and maximal thoracic aortic diameter in the total cohort and for males and females.

Variables	Absolute diameter (mm)				ASI (mm/m^2^)				AHI (mm/m)			
	Total (n = 181)	Male (n = 99)	Female (n = 82)	P-value	Total (n = 181)	Male (n = 99)	Female (n = 82)	P-value	Total (n = 181)	Male (n = 99)	Female (n = 82)	P-value
SoV	42.7 ± 6.2	41.1 ± 5.9	42.3 ± 6.4^2^	0.202	21.4 [16.8–24.5]	19.8 [17.2–22.2]^1^	23.8 [20.6–26.0]^2^	**<0.001**	23.9 ± 4.0	22.7 ± 3.5^1^	25.4 ± 4.0^2^	**<0.001**
AA	50.0 [46.0–55.0]	49.0 [45.0–53.0]	51.0 [47.0–57.0]	**0.023**	25.7 [22.6–29.7]	23.3 [21.2–25.8]^1^	29.4 [26.2–32.2]^2^	**<0.001**	28.9 [26.1–31.7]	27.2 [25.0–29.5]^1^	30.8 [28.5–33.7]^1^	**<0.001**
DA	34.0 [30.0–36.0]	33.0 [30.0–36.0]	34.0 [30.0–37.0]	0.454	16.9 [15.0–19.2]	15.8 [14.3–17.4]^1^	18.9 [16.6–20.9]^2^	**<0.001**	18.9 [16.7–21.2]	18.3 [16.3–19.8]^1^	20.1 [17.6–21.9]^1^	**<0.001**
ADmax	50.0 [47.0–56.0]	49.0 [46.0–54.0]	52.0 [47.0–57.0]	**0.027**	26.1 [23.0–30.0]	23.8 [21.7–26.3]^1^	29.7 [26.8–32.5]^2^	**<0.001**	29.2 [26.6–32.3]	27.4 [25.5–29.9]^1^	31.2 [28.9–34.0]^1^	**<0.001**

Normally distributed continuous variables are expressed as mean ± SD, skewed continuous variables are expressed as median and 25th-75th percentile. P-values < 0.05 are depicted in bold. Missing values are expressed with: ^1^:1%, ^2^: 2–5 % P-values are given for the comparison between males and females, P-values below 0.05 are depicted in bold. Abbreviations: ASI: aortic size index; AHI: aortic height index; SoV: Sinus of Valsalva; AA: ascending aorta; DA: descending aorta; ADmax: maximal aortic diameter.

**Fig. 3:**
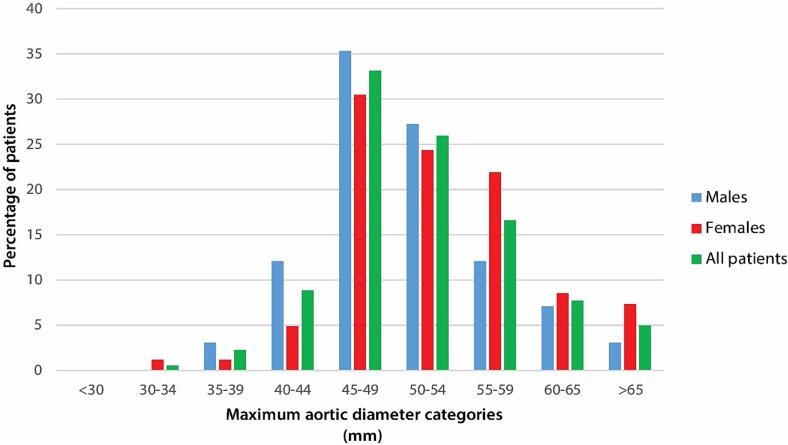
Percentages of maximal diameter categories at presentation for the whole cohort and males and females. P-value with Fisher exact test for all categories comparing males with females was 0.215.

The distribution of the absolute diameters, ASI and AHI at the three pre-defined levels for males and females are illustrated in Appendix V, Appendix VI and Appendix VII, respectively. In Fig. 3 the percentages of diameter categories are depicted for males, females and the total study population. The proportion of female patients with a diameter ≥55 mm was significantly higher than the proportion of male patients (n = 31/82, 37.8 % versus n = 22/99, 22.2 %, p = 0.022).

In Appendix VIII, the calculated Z-scores for the SoV and AA dimensions are shown. Female patients had significantly higher Z-scores than males at both levels (SoV: 2.99 ± 1.66 versus 1.34 ± 1.77, p < 0.001 and AA: 5.27 [4.38–6.26] versus 4.06 [3.14–5.02], p < 0.001).

### 3.3 Associations with maximal thoracic aortic diameter

Univariable and multivariable linear regression analyses for the log2-transformed ADmax are shown in Table 3. In univariable analysis, female sex was positively associated with the maximal thoracic aortic diameter (β: 0.070 (95 %CI 0.006–0.135), p = 0.032). None of the other included patient characteristics were significantly associated with ADmax in univariable analysis. After adjustment for age, history of hypertension and “known with thoracic aortic aneurysm”, female sex remained statistically significant (β: 0.079 (95 %CI 0.013–0.145), p = 0.019). After removal of influential observations, the univariable association between female sex and the ADmax remained significant (β: 0.063 (95 %CI 0.014–0.112), p = 0.011).

**Table 3:** Linear regression analysis for log transformed maximal thoracic aortic diameter.

Variable	Log2 max. thoracic aortic diameter			
	*Univariable analysis*		*Multivariable analysis*	
	Beta (95 %CI)	P value	Beta (95 %CI)	P value
Female sex *Male sex (ref)*	0.070 (0.006–0.135)	**0.032**	0.079 (0.013–0.145)	**0.019**
Age (*per one year increase)*	−0.002 (−0.004–0.001)	0.218	−0.002 (−0.005–0.001)	0.127
Height (m)	−0.301 (−0.617–0.015)	0.062	–	**–**
Weight (kg)	−0.001 (−0.003–0.001)	0.276	–	–
BSA (m^2^/mm)	−0.115 (−0.263–0.032)	0.124	–	–
History of hypertension	−0.013 (−0.079–0.053)	0.702	−0.021 (−0.088–0.046)	0.534
History of hyperlipidaemia	−0.058 (−0.151–0.035)	0.220	–	–
COPD	−0.055 (−0.176–0.066)	0.371	–	–
Prior CVA	−0.074 (−0.216–0.068)	0.303	–	–
Prior MI	0.121 (−0.044–0.286)	0.151	–	–
Chronic kidney disease	0.007 (−0.247–0.261)	0.955	–	–
BAV	0.007 (−0.173–0.187)	0.937	–	–
History of thoracic aortic aneurysm	0.040 (−0.066–0.145)	0.459	0.033 (−0.072–0.138)	0.138
Prior dissection or aneurysm in other major artery	−0.022 (−0.220–0.175)	0.823	–	–
Prior cardiac surgery	0.014 (−0.144–0.172)	0.863	–	–
Prior aortic surgery	−0.044 (−0.239–0.151)	0.654	–	–

Beta coefficients and corresponding 95 % CIs are shown. P-values < 0.05 are depicted in bold. Interpretation for beta coefficients: per one-unit increase of variable ×, y increases (i.e., the expected value of y will be multiplied with 2^beta^; eg, for female sex, the expected increase in maximal aortic diameter is 2^0.070^ (=1.050, 5.0 % increase).

“-“ means the variable is not included in the multivariable model.

Ref: reference; BSA: body surface area; COPD: chronic obstructive pulmonary disease; CVA: cerebrovascular accident; MI: myocardial infarction; BAV: bicuspid aortic valve.

## 4 Discussion

In this cross-sectional multicentre study on patients presenting with acute type A dissection (ATAAD), it was shown that females had larger absolute but especially indexed ascending aortic (AA) diameters and demonstrated to have larger aortic diameters when indexed for body surface area and height at the sinus of Valsalva (SOV), AA and descending aorta (DA). Furthermore, Z-scores for the SoV and AA were significantly larger in female patients. After adjustment for important clinical factors, female sex remained significantly associated with a larger maximal diameter.

The main finding of our study is the association of female sex with larger aortic dimensions at ATAAD presentation, even after adjusting for age, sex and body surface area using the Z-scores. The larger ascending aortic dimensions in female patients might reflect the older age as compared to male patients at time of ATAAD presentation. Interestingly, in Rylski et al. a greater diameter increase with age in the ascending aorta was observed in females compared to males in a healthy population [13]. This might be caused by a postmenopausal decrease in oestrogen in females, associated with an increased aortic wall stiffness [26]. Also, longitudinal studies on the natural history of thoracic aortic aneurysm patients show faster aneurysm growth in females than males [27], [28] and aortic stiffness was found associated with faster thoracic aortic aneurysm growth in females, yet not in males [28]. One also might speculate that male patients at high risk of aortic dissection already underwent preventive aortic surgery or reversely that females were less often diagnosed with aortic dilation female patients less often underwent surgery. In abdominal aortic aneurysm (AAA) patients, a meta-analysis showed that the proportion not-offered intervention was higher in females than in males [16], however this has not been investigated for thoracic aortic aneurysms. Based on our results, it can be argued that females dissect at larger diameters than males, and female sex is protective for aortic dissection. Possibly, the pre-menopausal phase is protective for women while at a certain postmenopausal age, changes in the aortic wall and increase in blood pressure attribute to greater aortic growth in females, warranting further research into male–female specific growth patterns.

In this study an association between age and smaller aortic diameters at time of ATAAD was observed, which has been described in other studies [29], [30]. Probably, some selection bias is present: as older patients with larger diameters might be underrepresented in the tertiary care centres in this study, and a relatively larger proportion of younger patients with hereditary thoracic aortic disease and larger diameters could have been included. It is well-known that many patients with ATAAD do not reach the hospital and the hospital-based population does not reflect the whole ATAAD population [2]. In a study comparing pre-dissection diameters with ATAAD diameters, patients with a pre-dissection scan were significantly older than those without [18]. It might be possible that older patients with thoracic aortic aneurysms and cardiovascular risk factors were under regular follow-up and ATAAD was prevented with an intervention or other medical treatment in these patients.

As mentioned in previous literature, the cut-off value of 55 mm for preventive surgery can be debated, as most aortic dissections occur at smaller diameters [29], [31] and pre-dissection diameters appear to be smaller [17], [18]. The mean absolute maximal diameter (52 ± 9) in the total study population was comparable to other studies with a mean of 53 ± 10 mm in Parish et al. [31], and a mean of 53 mm in Pape et al. [29]. Recently, Zafar et al. found that thoracic aortic aneurysm patients already had an increased risk for aortic complications at a diameter of 52.5 mm [32], unfortunately a male–female specific analysis was not provided. The current study shows comparable results but also draws special attention to the relatively smaller diameters observed in males: 62 % of females presented with a diameter below 55 mm compared to 78 % of males (p = 0.022). In contrast, the study by Parish et al. on ATAAD patients found comparable proportions between males and females: 59 % in females compared to 63 % in males (not statistically tested). These contrasting results might be explained by variation in study populations: patients with (suspected) Marfan syndrome, bicuspid aortic valve or previous cardiac surgery, were excluded in the study of Parish and colleagues [31]. In our study population the proportion of these patient groups was minimal. The latest American guidelines recommend intervention at a smaller absolute diameter in centers of expertise [8]. Since no differences between males and females were found in clinical outcomes after ascending aortic aneurysm surgery, although important male–female differences in pre- and perioperative characteristics were identified [33], this recommendation can be applied to both males and females based on the current evidence.

In clinical practice, the risk of aortic dissection or rupture, together with the risk of complications of preventive surgery and patient preferences should be carefully weighted. In our study, the ASI and AHI were larger for females compared to males at all levels in the thoracic aorta and Z-scores for the SoV and AA were larger in females. Although the Law of Laplace supports the use of the absolute diameter, research increasingly shows the value of the indexed thoracic aortic diameters in thoracic aortic aneurysm patients in risk stratification for aortic dissection or cardiovascular events [32], [34]. In female patients with Turner syndrome, who are known for their relatively small body height and increased risk of aortic complications, the ASI is already widely used [7], [8]. The use of Z-scores in risk stratification might be helpful especially in females, as it seems that some individuals have deviant Z-scores, while in absolute terms the aortic diameter is not (yet) enlarged. Also, increased awareness of aortic dilation in females is needed, because it is possible that females present at a more advanced disease stage or might be underdiagnosed. Early initiation of less invasive treatment options such as antihypertensive medication and lifestyle interventions need attention to prevent aneurysm growth.

Interestingly, only 11 % of patients was known with a thoracic aortic aneurysm in our study population. A meta-analysis on male–female differences in ATAAD, showed clear differences in patient characteristics and identified a female phenotype [35]. Future studies on male–female specific risk factors in population based studies and longitudinal studies measuring the aortic diameter are warranted to provide more insight into the risk profiles. Also, investigation of other metrics such as aortic length, peak wave velocity and blood biomarkers [36], [37], [38] can help unravel the pathophysiology behind the observed male–female differences.

## 5 Limitations

Some limitations to this study need to be mentioned. The most important limitation being that it is assumed that the increase in aortic diameter due to the impact of the dissection [17], [18] is comparable for males and females, however it is unknown whether these geometry changes are different. Measurement of the aortic diameter at time of dissection is known to be challenging. As the CT scans were mostly done in an emergency setting in referring centres, scanning protocols were different between patients. However, as the measurements in our study were performed by one observer using a pre-specified measuring protocol at specific anatomic levels, the measurements are considered reliable for the comparison between males and females. Furthermore, unfortunately only 59 % of ATAAD patients in a tertiary care setting had a CT scan eligible for the measurements, which might reduce the generalizability of our findings. The CT scans of referring centres were mostly missing due to technical problems with the transfer, making it impossible to import the scans in the measuring software system. In the sensitivity analysis comparing patients with and without CT scan no significant differences in patient characteristics were observed, so bias might be limited. Lastly, survival bias might be present: female or male ATAAD patients who did not reach the hospital, were not included in this study.

## 6 Conclusion

Our study showed that female patients with acute type A aortic dissection (ATAAD) had larger absolute aortic diameters at the level of the ascending aorta, whereas indexed aortic dimensions were larger at all three levels of the thoracic aorta compared to male patients. Also, Z-scores at the level of the sinus of Valsalva and ascending aorta were higher in female patients. These results imply a distinct presentation at ATAAD in the disease process for males and females or selection bias, warranting further male–female specific research into risk factors and growth patterns. Both males and females could benefit from a more personalized approach in preventive measures and risk stratification during follow-up.

## Author’s contributions

Study design: FM, WK, AG, CT, HT, JR-H. Data collection: FM, AB, WK, AG, CT. Data analysis: FM, AB. Data interpretation: all authors. Manuscript draft: FM, AB. Critical revision, editing and approval of the final manuscript: all authors.

## Declaration of Competing Interest

The authors declare that they have no known competing financial interests or personal relationships that could have appeared to influence the work reported in this paper.
