# Supplementary material for: Male-female differences in thoracic aortic diameters at presentation of acute type A aortic dissection
Source: Int J Cardiol Heart Vasc. 2023 Oct 29;49:101290. doi: 10.1016/j.ijcha.2023.101290 (PMC10628350; doi:10.1016/j.ijcha.2023.101290)
Supplement: Supplementary Data 1 [file mmc1.docx]

**Supplementary Material**

**Appendix I -** Sample size calculation

A sample size calculation was performed based on the estimates of the ascending aortic diameter in males and females in the Dutch general population of the study by Bons et al. using a web-based calculator: <https://statulator.com/SampleSize/ss2M.html>

The ascending aortic diameters were 38 ± 4 mm (n= 1208) for males and 35 ± 3 mm for females (n=1297). The pooled standard deviation was: 4 mm (Bons et al.).

The settings for the calculation were the default: a desired power of 0.80, level of significance 0.05, alternate hypothesis two-sided. As the male-female ratio in acute type A aortic dissection is estimated at 3:2, the ratio of the reference to the test group was set at 1.5.

This resulted in an estimated sample size of 24 females and 36 males (total 60).

**Appendix II –** Definitions of variables

| Variables | Unit | Definition |
| --- | --- | --- |
| *Patient demographics* |  |  |
| Age | Years | Age at presentation calculated from date of birth |
| Sex |  | As reported in patient file |
| Body mass index |  | $\frac{Weight (kg)}{{Height}^{2} (m2)}$ |
| Body surface area |  | Dubois-Dubois formula  $({Weight}^{0.425})*({Height}^{0.725})*0.007184$ |
| History of hypertension |  | Medical treatment for hypertension or described in patient history |
| Hyperlipidaemia |  | Medical treatment for hyperlipidaemia or described in patient history |
| Diabetes mellitus |  | Medical treatment for diabetes mellitus or described in patient history |
| Chronic obstructive pulmonary disease (COPD) |  | Any history of COPD that required medical treatment or FEV1<70%) |
| History of cerebrovascular disease (CVA) |  | History of TIA (transient ischemic attack) or stroke in patient history |
| History of myocardial infarction (MI) |  | As described in patient history |
| Chronic kidney disease |  | As described in patient history |
| Heritable thoracic aortic disease |  | History of a known heritable thoracic aortic disease as diagnosed before or after ATAAD presentation. In this variable missing values were not included, i.e. if it was unknown, HTAD status was considered as “no”. |
| Known TAA prior to presentation |  | Thoracic aorta > 40 mm |
| Smoking (never, currently, past) |  | As described in the patient history |
| Prior cardiac surgery |  | As described in the patient history |
| Prior aortic surgery |  | As described in the patient history |
| Prior dissection or aneurysm in other major artery |  | History of a dissection or aneurysm in another vessel excluding the thoracic aorta |
| BAV |  | Bicuspid aortic valve as diagnosed during surgery |
| History of AS |  | History of aortic valve stenosis >grade 2 |
| History of AR |  | History of aortic valve regurgitation >grade 2 |
| *CT-imaging characteristics* |  |  |
| Contrast-enhanced |  | Contrast enhanced scan |
| ECG-triggered |  | ECG-triggered multiphase CT scanning |
| Dissected AR |  | Dissected aortic root as observed at scan |
| Dissected AA |  | Dissected ascending aorta as observed at scan |
| Dissected DA |  | Dissected descending aorta as observed at scan |
| Absolute diameter | mm | Absolute aortic diameter |
| Aortic size index | mm/m^2^ | Absolute aortic diameter divided by body surface area |
| Aortic height index | mm/m | Absolute aortic diameter divided by body height |

Abbreviations: BMI: body mass index; BSA: body surface area; MI: myocardial infarct; TAA: thoracic aortic aneurysm; CABG: coronary artery bypass graft; PCI: percutaneous coronary intervention; BAV: bicuspid aortic valve; AS: aortic valve stenosis; AR: aortic regurgitation; CT: cardiac tomography; ECG: electrocardiography; AA: aortic root, AA: ascending aorta, DA: descending aorta.

| **Appendix III –** Sensitivity analysis: CT-scan versus no CT-scan | | | |  |
| --- | --- | --- | --- | --- |
|  | **CT-scan**  **(n=181)** | **No CT-scan (n=127)** | **P-value** | **Missing**  **n (%)** |
| **Age (years)** | 62 ± 13 | 62 ± 12 | 0.697 | 0 (0) |
| **Females (%)** | 82 (45) | 53 (42) | 0.534 | 0 (0) |
| **Height (m)** | 1.75 ± 0.10 | 1.75 ± 0.10 | 0.857 | 7 (2) |
| **Weight (kg)** | 80.3 ± 15.2 | 81.0 ± 16.6 | 0.723 | 8 (3) |
| **BMI (kg/m^2^)** | 25.6 [23.4; 28.1] | 26.0 [23.1; 28.8] | 0.455 | 9 (3) |
| **BSA (m^2^)** | 1.97 ± 0.22 | 1.98 ± 0.24 | 0.783 | 9 (3) |
| **History of hypertension (%)** | 99 (55) | 71 (57) | 0.796 | 4 (1) |
| **Hyperlipidemia (%)** | 25 (15) | 16 (13) | 0.703 | 11 (3) |
| **Diabetes Mellitus (%)** | 3 (2) | 1 (1) | 0.674 | 8 (3) |
| **COPD (%)** | 14 (8) | 12 (10) | 0.585 | 4 (1) |
| **History of CVA (%)** | 10 (6) | 4 (3) | 0.329 | 4 (1) |
| **History of MI (%)** | 7 (4) | 4 (3) | 1.000 | 2 (1) |
| **Chronic kidney disease (%)** | 3 (2) | 3 (2) | 0.694 | 5 (1) |
| **Heritable thoracic aortic disease (%)*** | 5 (4) | 3 (4) | 0.593 | 116 (38) |
| **Known TAA prior to presentation (%)** | 19 (11) | 11 (9) | 0.591 | 5 (1) |
| **Smoking (%)**   - **Never** - **Currently** - **Past** | 41 (38)  45 (42)  22 (20) | 29 (43)  27 (40)  11 (17) | 0.721 | 133 (43) |
| **Prior cardiac surgery (%)^3^** | 8 (4) | 3 (2) | 0.639 | 6 (2) |
| **Prior aortic surgery (%)** | 5 (3) | 2 (2) | 0.645 | 0 (0) |
| **Prior dissection or aneurysm in other major artery (%)** | 5 (3) | 2 (2) | 0.704 | 3 (1) |
| **History of AS (%)** | 2 (3) | 1 (5) | 0.564 | 216 (70) |
| **History of AR (%)** | 4 (6) | 3 (14) | 0.352 | 216 (70) |

Normally distributed continuous variables are expressed as mean ± SD, skewed continuous variables are expressed as median and 25th-75th percentile, and categorical values are expressed as percentages. P-values < 0.05 are depicted in bold. Missings are expressed as the absolute number and the represented percentages in brackets. *Patients were diagnosed with heritable thoracic aortic disease after ATAAD presentation and included Loeys-Dietz syndrome (n=1), ACTA2 mutation (n=1), Marfan syndrome (n=1), a variant of unknown significance in the TGB3 gene (n=1) and the MYLK gene (n=1), respectively.

Abbreviations: BMI: body mass index; BSA: body surface area; MI: myocardial infarction; TAA: thoracic aortic aneurysm; CABG: coronary artery bypass graft; PCI: percutaneous coronary intervention; AS: aortic valve stenosis; AR: aortic regurgitation

**Appendix IV -** Sensitivity analysis: excluding patients with bicuspid aortic valves and heritable thoracic aortic disease diagnosed after acute type A aortic dissection. Absolute, BSA-indexed and height-indexed diameters of the Sinus of Valsalva, ascending aorta, descending aorta and maximal thoracic aortic diameter in the total cohort and for males and females

|  | Absolute diameter (mm) | | | | | ASI (mm/m^2^) | | | AHI (mm/m) | | | | | |
| --- | --- | --- | --- | --- | --- | --- | --- | --- | --- | --- | --- | --- | --- | --- |
| Variables | **Total**  **(n=170)** | **Male**  **(n=95)** | **Female**  **(n=75 )** | **P-value** | **Total (n=170)** | | **Male**  **(n=95)** | **Female**  **(n=75)** | | **P-value** | **Total (n=170)** | **Male**  **(n=95)** | **Female**  **(n=75)** | **P-value** |
| Sinus of Valsalva | 41.5 ± 6.2 | 41.0 ± 5.9 | 42.3 ± 6.5 | 0.178 | 20.85 [18.6-24.3] | | 19.7  [17.2-22.0] | 23.9  [20.8-26.0] | | **<0.001** | 23.8 ± 4.0 | 22.5 ± 3.43 | 25.4 ± 4.1 | **<0.001** |
| Ascending aorta | 49.0  [46.0-54.8] | 49.0  [45.0-53.5] | 50.0  [47.0-55.5] | **0.049** | 25.7  [22.6-29.7] | | 23.3  [21.3-25.7] | 29.4  [26.4-32.2] | | **<0.001** | 28.8  [26.1-31.6] | 27.2  [25.1;29.4] | 30.7  [28.6-33.5] | **<0.001** |
| Descending aorta | 34.0  [30.0-36.0] | 34.0  [30.0-36.0] | 34.0  [30.0-37.5] | 0.405 | 16.9  [14.9-19.3] | | 15.8  [14.4-17.3] | 19.1  [16.8-21.0] | | **<0.001** | 18.9  [16.6-21.2] | 18.3  [16.2;19.5] | 20.5  [17.7-22.3] | **<0.001** |
| Maximal thoracic aortic diameter | 50.0  [47.0-55.0] | 49.0  [46.0-54.0] | 51.0  [47.0-56.0] | **0.033** | 26.0  [22.9-30.0] | | 23.6  [21.7-26.0] | 29.7  [26.9-32.5] | | **<0.001** | 29.1  [26.5-31.9] | 29.1  [26.5;31.9] | 31.2  [29.1-33.9] | **<0.001** |

Normally distributed continuous variables are expressed as mean ± SD, skewed continuous variables are expressed as median and 25th-75th percentile. P-values < 0.05 are depicted in bold. Missing values are expressed with: ^1^:1%, ^2^: 2-5% P-values are given for the comparison between males and females, P-values below 0.05 are depicted in bold. Abbreviations: BSA: body surface area; ASI: aortic size index; AHI: aortic height index. Patients of which bicuspid aortic valve or heritable thoracic aortic disease disease was not known, were considered as “no” in this analysis.

**Appendix V** - Distribution of absolute aortic diameters for males and females.


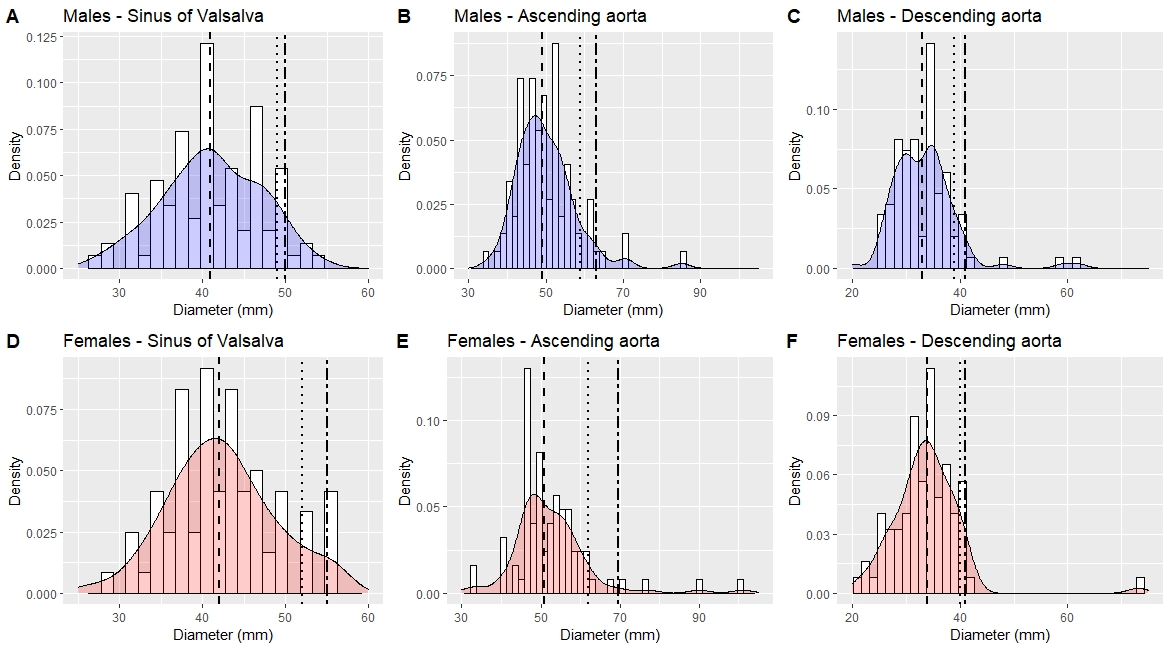


Panel a, b and c represent the distribution for the males and panel d, e and f represent the distribution for the females. In addition to the distribution curve, the median, 90^th^ and 95^th^ percentile, respectively as the first, second and third striped vertical lines, are visualized.

**Appendix VI** - Distribution of aortic size index (ASI) for males and females.


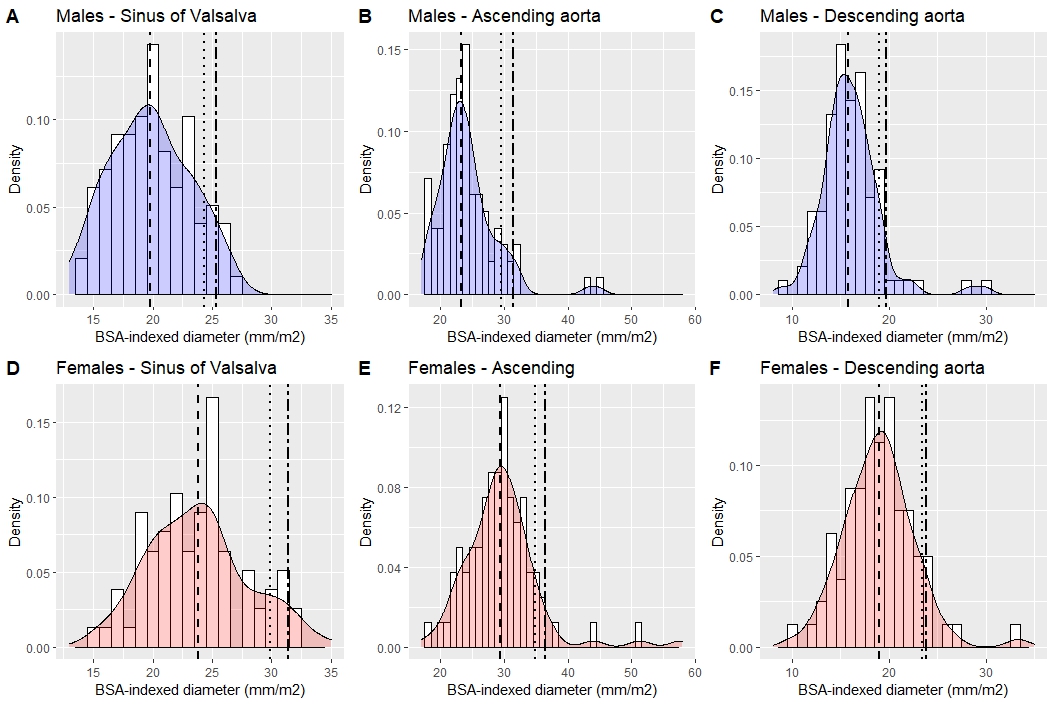


Panel a, b and c represent the distribution for the males and panel d, e and f represent the distribution for the females. In addition to the distribution curve, the median, 90^th^ and 95^th^ percentile, respectively as the first, second and third striped vertical lines, are visualized.

**Appendix VII** - Distribution of aortic height index (AHI) for males and females.


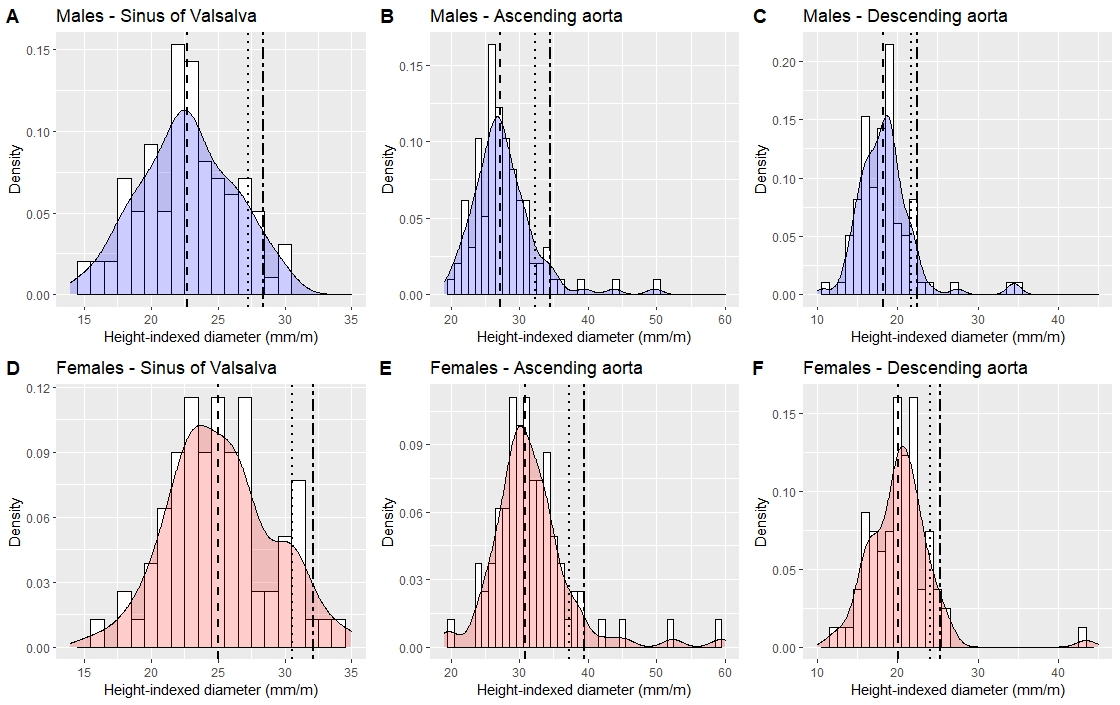


Panel a, b and c represent the distribution for the males and panel d, e and f represent the distribution for the females. In addition to the distribution curve, the median, 90^th^ and 95^th^ percentile, respectively as the first, second and third striped vertical lines, are visualized.

**Appendix VIII** – Z-scores for the whole study population and for males and females separately.

|  | Total (n=181) | Male (n=99) | Female (n=82) | P-value | Missing |
| --- | --- | --- | --- | --- | --- |
| Sinus of Valsalva (SoV) | 2.07 ± 1.90 | 1.34 ± 1.77 | 2.99 ± 1.66 | <0.001 | 2.8 |
| Z-score SoV > 2.00 | 95 (54.0) | 37 (37.8) | 58 (74.4) | <0.001 | 2.8 |
| Ascending aorta  (AA) | 4.56  [3.70-5.65] | 4.06  [3.14-5.02] | 5.27  [4.38-6.26] | <0.001 | 1.7 |
| Z-score AA > 2.00 | 171 (96.1) | 93 (94.9) | 78 (97.5) | 0.461 | 1.7 |

The Z-score calculated using the Campens formula is depicted as the mean with standard deviation for the SoV and median and 25^th^-75^th^ percentile for the AA. The proportion of patients with a Z-score > 2.00 is shown as percentage.
